# Interviewing autistic adults: Adaptations to support recall in police, employment, and healthcare interviews

**DOI:** 10.1177/1362361320909174

**Published:** 2020-03-23

**Authors:** Jade Eloise Norris, Laura Crane, Katie Maras

**Affiliations:** 1 University of Bath, UK; 2 University College London, UK

**Keywords:** autism, criminal justice system, employment, episodic, healthcare, interviewing, memory, preparation, recall, task support

## Abstract

Recalling specific past experiences is critical for most formal social interactions, including when being interviewed for employment, as a witness or defendant in the criminal justice system, or as a patient during a clinical consultation. Such interviews can be difficult for autistic adults under standard open questioning, yet applied research into effective methods to facilitate autistic adults’ recall is only recently beginning to emerge. The current study tested the efficacy of different prompting techniques to support autistic adults’ recall of specific personal memories; 30 autistic and 30 typically developing adults (intelligence quotients > 85) were asked to recall specific instances from their past, relevant to criminal justice system, healthcare, and employment interviews. Questions comprised ‘open questions’, ‘semantic prompting’ (where semantic knowledge was used to prompt specific episodic retrieval) and ‘visual–verbal prompting’ (a pie-diagram with prompts to recall specific details, for example, who, what, and where). Half the participants received the questions in advance. Consistent with previous research, autistic participants reported memories with reduced specificity. For both groups, visual–verbal prompting support improved specificity and episodic relevance, while semantic prompting also aided recall for employment questions (but not health or criminal justice system). Findings offer new practical insight for interviewers to facilitate communication with typically developing and autistic adults.

**Lay abstract:**

During many types of interviews (e.g. in employment, with the police, and in healthcare), we need to recall detailed memories of specific events, which can be difficult for autistic people in response to commonly used questions. This is especially because these tend to be open questions (i.e. very broad). Autistic people have disproportionately high rates of physical and mental health conditions, are more likely to interact with police, and are the most underemployed disability group. However, interviewers are often unsure about how to adapt their communication for autistic people.

Our research tested whether different types of prompts enabled autistic people to recall specific memories (memories of a single event within one day). Participants were asked about situations relating to witnessing a crime (e.g. at the bank), physical or mental health scenarios and employment interviews (e.g. a time you’ve met a deadline).

We tested the following:

- *Open questions*: basic questions only (e.g. ‘tell me about a time you went to the cinema’),
- *Semantic prompting*: a general prompt (e.g. ‘do you enjoy going to the cinema?’) before asking for a specific instance (‘tell me about a time you went to the cinema?’),
- *Visual–verbal prompting*: asking participants to recall *when* it happened, *who* was there, the *actions* that occurred, the *setting*, and any *objects*.

With visual–verbal prompting, autistic and typically developing participants’ memories were more specific and detailed. Semantic prompting was also effective for employment questions. Our study shows that autistic people can recall specific memories when they are appropriately prompted. Visual–verbal prompting may be effective across different situations.

Autobiographical memories (ABMs) comprise both personally experienced events (‘personal episodic memories’, for example, my first day at school) and facts related to the self (‘personal semantic memories’, for example, I used to live in London). Recalling specific ABMs that happened on one particular day, at a specific place and time (Conway & Rubin, 1993; Piolino et al., 2010) aids a range of everyday and formal situations. In the criminal justice system (CJS), for example, an eyewitness who provides an elaborate, detailed account of an incident is likely to offer more investigative leads than an eyewitness whose account is lacking specificity, is deficient in contextual details or deviates from a temporal account (Gaigg & Bowler, 2018). Similarly, in healthcare consultations, providing specific information about the onset of an illness or injury can be crucial in supporting a clinical diagnosis (e.g. taking a history, reporting symptom onset and what makes them better/worse), while evidencing claims about possessing favourable skills and experience with specific examples is important for success in employment interviews (Barclay, 2001; Campion et al., 1988).

Autistic people often experience difficulties in recalling specific personal episodic memories (Ben Shalom, 2003; Crane & Goddard, 2008; Goddard et al., 2007; Klein et al., 1999; McDonnell et al., 2017). These difficulties are characterised by over-general recollection, with autistic adults retrieving fewer or less specific memories, and taking significantly longer to do so (see Crane & Maras, 2018; Gaigg & Bowler, 2018). This is particularly pertinent for CJS, health and employment interviews because autistic individuals currently face significant disadvantages in each of these areas. Due to factors such as social vulnerability and difficulty with understanding others’ intentions, autistic people are more likely to be questioned in the CJS (e.g. Chaplin & Mukhopadhyay, 2018; Rava et al., 2017; Tint et al., 2017; Weiss & Fardella, 2018), yet current interviewing techniques are ineffective in eliciting their best evidence (see Maras, in press; Maras & Bowler, 2014). Autistic people also experience significantly higher rates of physical and mental health problems (Bishop-Fitzpatrick & Kind, 2017; Croen et al., 2015), yet struggle with accessing appropriate healthcare given their communication needs (Mason et al., 2019; Muskat et al., 2015; Nicolaidis et al., 2015; Raymaker et al., 2017). Regarding employment, 85% of autistic people are not in full-time work (Knapp et al., 2009; see also Gotham et al., 2015; Hendricks, 2010; Howlin, 2013; Levy & Perry, 2011; Lounds-Taylor et al., 2015; Shattuck et al., 2012), and around 46% of employed autistic adults are over-educated or over-skilled for their current role (Baldwin et al., 2014). Interviews have been reported to be a major barrier to gaining employment (Scott et al., 2019).

A common factor across these contexts is the use of open questions (e.g. ‘tell me what happened at the crime scene’, ‘tell me about your accident’, ‘tell me about a time you’ve met a deadline’; Conway & Peneno, 1999; Gask & Usherwood, 2002; Ministry of Justice, 2011). Yet, this style of questioning is problematic for autistic people, whose performance usually becomes more impeded relative to typically developing (TD) individuals the greater the open-ended nature of the task (see, for example, Gaigg & Bowler, 2018; Maras, in press). This may be due to difficulties with theory of mind and forming an implicit understanding of the questioner’s expectations (e.g. Kenworthy et al., 2008; White, 2013, see also Milton, 2012), coupled with executive processing demands (Maister et al., 2013) and relational processing difficulties (see Bowler et al., 2009; Gaigg & Bowler, 2018).

Critically, task support in the form of cued recall or recognition tests has been shown to improve autistic individuals’ recall of past events compared to free recall (e.g. Bowler et al., 1997, 2004, or see Boucher et al., 2012). The ‘Task Support Hypothesis’ posits that, with more specific and supportive cues, autistic people can recall as much information as TD peers (Bowler et al., 1997, 2004). The use of support, such as asking specific questions, can reduce error reporting (e.g. Maras et al., 2013) and increase the amount of accurate information reported (e.g. Almeida et al., 2019; Mattison et al., 2015, 2018).

The provision of more support at test may also facilitate the *relevance* of responses. Indeed, autistic people sometimes provide fewer relevant and more irrelevant details in their recall of events. For example, on a semi-structured conversation narrative recall task, Losh and Gordon (2014) found that autistic participants produced more off-topic and irrelevant remarks, departed from the main story themes and produced less coherent stories. In line with the task support hypothesis, these differences in performance were reduced on a structured story task that involved narrating from a wordless picture book. This indicates that the provision of cues can reduce the ambiguity of what is required by a task, and help to control attention and facilitate the organisation of recall (Losh & Gordon, 2014; see also Losh & Capps, 2003).

A further, but as yet untested, avenue for supporting autistic individuals’ episodic ABM retrieval involves first drawing upon semantic ABM first as a cue to elicit specific ABMs. Robinson et al. (2017) found that autistic participants (aged 11–18 years) recalled significantly fewer of their own personality traits, but a similar number and type of specific episodic memories to TD individuals (although they required more initial prompts to do so). Whilst this finding is in contrast to previous literature (e.g., Crane & Goddard, 2008; Crane et al., 2009), the authors suggested that this may be due to the task structure: initially requesting semantic knowledge about the self may have drawn upon intact semantic ABM structures to scaffold the retrieval of specific memories. This is consistent with Conway and Pleydell-Pearce’s (2000) proposal that ABM is organised hierarchically, with cues first activating general memories (e.g. ‘studying at university’), followed by more specific exemplars (e.g. ‘my first day at university’). When retrieving specific memories, an individual must inhibit each inappropriate general memory encountered during the retrieval search in order to focus on a specific exemplar. This is coordinated by a component termed the ‘working self’ (a direct analogy with Baddeley’s, 1986, model of working memory) that arranges memories into goal hierarchies according to the current self-concepts (see also Dalgleish et al., 2007). This is of relevance to autistic people because the difficulties they experience in recalling specific ABMs have been suggested to be related to problems in using the self as an effective memory organisation system (Crane et al., 2009, 2010). Furthermore, the executive functioning difficulties often reported in autism (Demetriou et al., 2018; Hill, 2004) have been implicated in autistic children’s and adults’ specific ABM retrieval difficulties (Crane et al., 2009; Crane & Goddard, 2008; Goddard et al., 2014). Thus, tasks which draw upon intact semantic processing (e.g. Crane et al., 2009) may reduce executive processing demands and scaffold episodic memory (e.g. Miller et al., 2014).

Allowing time for preparation may also benefit autistic people’s recall and is championed by autistic people as a key strategy used to minimise anxiety caused by unpredictable events (Robertson et al., 2018). Autistic individuals have been reported to perform as well as TD individuals on written and online tasks (Crane et al., 2013; Zamoscik et al., 2016), which may represent a less stressful retrieval context wherein social demands are not present. Employment experts are increasingly advocating for providing candidates with interview questions in advance to ensure that assessment is based on work history and skills, rather than presentation performance (particularly for disabled groups; Jordan, 2008). Furthermore, healthcare patients are advised to prepare for doctor’s appointments by making notes (The Patients Association, n.d.), while witness familiarisation courses in England and Wales aim to prepare witnesses for court by familiarising them with the environment and court procedures, during which questioning techniques used by lawyers during cross-examination may also be discussed (Wheatcroft, 2017; Wheatcroft & Ellison, 2012).

In sum, constructing an appropriately detailed, relevant, and coherent-free narrative requires retrieving a specific past event and generating, monitoring and controlling output while considering the listener’s perspective. These are all areas of difficulty for an autistic person (see Maras, in press). Autistic people may need guided retrieval from the outset to (a) support memory retrieval, (b) reduce implicit social demands regarding relevance and (c) minimise demands on executive resources. The primary aim of the current study was to test the effectiveness of two novel supportive questioning techniques, ‘Semantic Prompting’ (using initial semantic prompts to elicit subsequent episodic retrieval) and ‘Visual–Verbal Prompting’ (V-VP; providing verbal and visual cues to indicate which aspects of the memory to report), against standard open questions in eliciting specific and relevant memories from autistic adults, across topics relevant for CJS, healthcare and employment interviews. The secondary aim was to examine the effect of providing preparation, whereby participants received the questions in advance and could write notes. It was predicted that with open questions, autistic participants would recall less specific memories than TD comparison participants, with fewer relevant episodic details, and more irrelevant details, but that differences would diminish with semantic prompting, V-VPs and preparation.

## Method

### Participants

In total, 30 autistic participants (17 males, 11 females, 2 other: genderfluid and no gender preference) and 30 TD participants (8 males, 22 females) took part. Participants were recruited primarily from the South West of England, including via social media, support groups and local community recruitment (posters, magazine articles, etc.). All autistic participants had received a formal clinical diagnosis of autism spectrum disorder (ASD) according to *Diagnostic and Statistical Manual of Mental Disorders* criteria (American Psychiatric Association, 2013), and confirmed this with a copy of their diagnostic report. Those who had received a diagnosis but were unable to access their report received the *Autism Diagnostic Observation Schedule, Second Edition* (ADOS-2; Lord et al., 2012), to confirm the diagnosis. Autistic and TD groups were matched on verbal intelligence quotient (IQ), *t*(58) = −0.77, *p* = .446, *d* = 0.20, and age, *t*(58) = −0.57, *p* = .574, *d* = 0.15, and did not significantly differ on Performance IQ or Full-Scale IQ (all *p*s > .051; see Table 1). A series of 2 (Group) × 2 (Prep) analyses of variance (ANOVAs) confirmed that the autistic and TD Prep versus No Prep groups did not differ on verbal IQ (VIQ) (*F*s < 0.62, *p*s > .435, $\eta_{p}^{2}s$ < .01), performance IQ (PIQ) (*F*s < 4.00, *p*s > .050, $\eta_{p}^{2}s$ < .08), full-scale IQ (FSIQ) (*F*s < 3.22, *p*s > .078, $\eta_{p}^{2}s$ < .05) and age (*F*s < 0.31, *p*s > .580, $\eta_{p}^{2}s$ < .01). All TD participants scored below the recommended minimum cut-off of 32 on the autism spectrum quotient (AQ-50 with 80% specificity; Baron-Cohen et al., 2001). The autistic group scored significantly higher on the AQ than the TD group, *t*(57) = −9.26, *p* < .001, with 18 scoring above the recommended minimum cut-off of 32 (Table 1). Ethical approval was obtained from the Psychology Research Ethics Committee at the University of Bath.

**Table 1.** Mean age, WASI-II, and AQ scores by group (standard deviations in parentheses).

	TD adults (*n* = 30^a^)	Autistic adults (*n* = 30)
Age (years)	34.87 (13.08); range = 18–59	33.00 (12.02); range = 18–58
VIQ	108.83 (8.38); range = 94–142	106.97 (10.05); range = 85–128
PIQ	113.70 (10.75); range = 92–136	107.50 (12.84); range = 82–131
FSIQ	112.63 (7.21); range = 95–126	108.17 (11.08); range = 89–129
AQ-50	13.97 (8.56); range = 2–30	34.90 (8.80); range = 14–48

AQ: autism spectrum quotient; WASI-II: Wechsler Abbreviated Scale of Intelligence; TD: typically developing; VIQ: verbal IQ; PIQ: performance IQ; FSIQ- full-scale IQ.

a AQ data for one autistic participant were not available.

### Design

The study utilised a 2 (Group: autistic vs TD) × 2 (Prep: preparation vs no preparation) × 3 (Support: open vs semantic prompting vs V-VP) × 3 (Context: CJS vs health vs employment) mixed factorial design, where support and context were within subjects. To minimise carry-over effects of support, conditions were administered in a fixed order (consistent with Crane et al., 2012; Piolino et al., 2010): (a) open questions, (b) semantic prompting and (c) V-VP.

### Measures

#### ABM questions

The study utilised an ABM interview task comprising questions about specific instances of potential witness scenarios in the CJS (where crimes may take place; for example, ‘tell me about a specific time . . . when you went to the bank’), physical or mental health scenarios (e.g. ‘tell me about a specific time . . . when you vomited’) and social and non-social scenarios relevant to employment (e.g. ‘tell me about a specific time . . . when you’ve shown someone how to use a piece of technology/met a deadline’). The interview comprised 18 questions (six CJS, six health and six employment) and was developed specifically for the study, building on Crane and Goddard’s (2008) ABM interview (see also Bekerian et al., 2001). Questions were refined following an online survey conducted with 95 TD and 26 autistic people (including two respondents with an informal diagnosis and two awaiting a formal diagnosis) to ensure that the questions overall represented situations that were not disproportionately more common for one group than the other.^1^

The 18 interview questions were split between the three different support conditions, resulting in six questions in total per support condition (two from the CJS context, two from health and two from employment; see Supplementary Materials A for full list). Questions were balanced within each Support × Context condition according to the type of event such that (in all three of the support conditions) for the CJS context, one question related to places and one to events; in the employment context, one question related to social and one to non-social work tasks; and in the health context, one question related to mental health and one to physical health.

##### Question support

Open questions provided no support (i.e. ‘tell me about a time . . .’), while semantic prompting used an initial prompt to cue semantic ABM (e.g. ‘do you enjoy going to the cinema?’) before then asking for a relevant specific instance in an identical format to the open questions (e.g. ‘tell me about a time when you went to the cinema?’). Finally, V-VP support (adapted from Brown & Pipe, 2003; ‘Verbal Labels’) also involved asking the initial question in open-question format immediately followed by further instruction about the details that were expected (‘tell me about when it happened, the people who were there, the actions that occurred, the setting, and the objects that were there’; see Table 2). Participants also received a paper copy of the V-VPs ‘wheel’ prompt, and a coin to use to keep track as they moved between the words (in any order).

**Table 2.** Example support adaptations for questions within the employment context.

Support	Example questions	
Open questions	‘Tell me about a specific instance, more than a week ago, when you have had to make a difficult decision’.	
Semantic prompting	‘Are you good at organising things?’ (respondent answers). ‘Tell me about a specific instance, more than a week ago, when you have organised something’.	
V-VP	‘Tell me about a specific instance, more than a week ago, when you have met a deadline. Tell me about *when* it happened, the *people* who were there, the *actions* that occurred, the *setting*, and the *objects* that were there. You should use this card to help you structure your answer’.	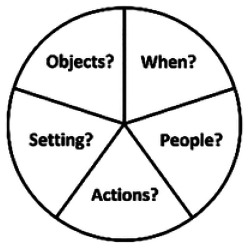

V-VP: visual–verbal prompting.

##### Preparation

Participants were randomly assigned to receive the questions in advance (‘Prep’) or not (‘No Prep’). Participants receiving preparation were given a summary of the task instructions and the question topics in open-question format (they were not informed about support) and a visual schedule (details about the appointment, including a photo of the researcher and the room). They were also encouraged to make notes and bring these to use during the interview. Participants were asked to read the preparation materials and think of their memories by themselves, and were advised that they should not seek help from others while doing this. Participants not receiving preparation were not given specific information regarding what they would be asked about prior to the appointment, but were fully informed about the study and told that they would be asked to recall memories of personally experienced events.

### Procedure

All participants received detailed instructions regarding what was expected of them during the interview, including that they should recall a specific memory for each question, defined as a particular event from more than a week ago (due to the tendency for people to recall more recent events, which tend to be more specific; Jansari & Parkin, 1996) lasting no longer than a day (Williams & Broadbent, 1986). All participants received instructions as to the level of detail expected, an example of a specific memory, and a paper summary of the instructions (see Supplementary Materials B and C).

#### Prompting

If participants gave no reply, a very limited response or only semantic/general information, the interviewer prompted them up to once per question: ‘Can you think of a particular time, within a 24 hour period? One specific instance?’ (Crane et al., 2012). If they recalled the same event more than once to different questions during the interview, the interviewer asked them to recall a new memory. Interviews lasted on average 57 min (*SD* = 23, range = 21–132 min),^2^ were audio recorded and transcribed verbatim.

At the end of the interview, participants were asked which aspects they found difficult/easy, whether they preferred a question type and (for those receiving prep) whether preparation was helpful (see Supplementary Materials D).

#### Coding

Transcripts were imported into NVivo (2012) where responses to each question were coded for overall specificity, and then each unit of information provided was coded as episodic versus semantic and relevant versus irrelevant. In order to accurately measure the effect of support (semantic prompting and V-VPs) compared to open questions with no support, only details given by participants *prior* to a generic prompt were coded^3^ (see Supplementary Materials G for analyses, including responses after the prompt); 47% of the transcripts were double-coded, with good interrater reliability for specificity (*r* = .728, α = .873) and relevance (episodic relevant, *r* = .961, α = .801; episodic irrelevant, *r* = .742, α = .938; semantic relevant, *r* = .829, α = .766; semantic irrelevant, *r* = .683, α = .556), *p*s < .001. In cases of disagreement, the first author’s ratings were analysed.

##### Specificity

Participants’ responses to each question were coded for level of specificity on a 5-point scale (Piolino et al., 2002; see Supplementary Materials E).

##### Episodic and semantic relevance

For each response, each new unit of information was coded as episodic or semantic, and as relevant or irrelevant. Episodic details were coded as relevant when they directly related to the temporal event (e.g. feeling cold during that particular supermarket visit) as well as episodic details directly related to the specific instance being discussed (e.g. referring to the outcome of a previous doctor’s appointment). Any episodic details about unrelated events were coded as irrelevant (e.g. discussing a later cinema trip in response to a question about going to the supermarket). Semantic information referring to general, non-event-specific information was coded as relevant (e.g. general time management skills when discussing meeting a deadline) or irrelevant (not related to the question, or referring to another person, for example, their father’s poor time management skills; see Supplementary Materials F for an example-coded response).

## Results

All mixed factorial ANOVAs were conducted as 2 (Group: autistic vs TD) × 2 (Prep: prep vs no prep) × 3 (Support: Open vs Semantic Prompting vs V-VP) × 3 (Context: CJS, health, employment), with support and context within subjects. Where the assumption of sphericity was violated, Greenhouse–Geisser corrections were applied.

### Specificity

Overall, autistic participants produced memories with lower specificity (*M* = 3.22, *SD* = 0.50) compared to TD participants (*M* = 3.50, *SD* = 0.41), *F*(1, 56) = 5.72, *p* = .020, $\eta_{p}^{2}$ = .09. There was a main effect of Support, *F*(2, 112) = 19.34, *p* < .001, $\eta_{p}^{2}$ = .26, with pairwise comparisons indicating significantly higher specificity in response to V-VP (*M* = 3.56, *SD* = 0.43) compared to both open questions (*M* = 3.24, *SD* = 0.57; *p* < .001, *d* = 0.63) and semantic prompting (*M* = 3.26, *SD* = 0.58; *p* < .001, *d* = 0.59), with no significant difference between the latter two (*p* = .696, *d* = 0.03). There was also a main effect of Context, *F*(2, 112) = 51.16, *p* < .001, $\eta_{p}^{2}$ = .48. Pairwise comparisons indicated that specificity was higher in response to questions in the CJS context (*M* = 3.66, *SD* = 0.41) than health (*M* = 3.31, *SD* = 0.59; *p* < .001, *d* = 0.69) and employment (*M* = 3.10, *SD* = 0.57; *p* < .001, *d* = 1.13), with the health context also yielding higher specificity than employment (*p* = .001, *d* = 0.36). There was no main effect of Prep, *F*(1, 56) = 0.55, *p* = .460, $\eta_{p}^{2}$ = .01.

**Figure 1.**
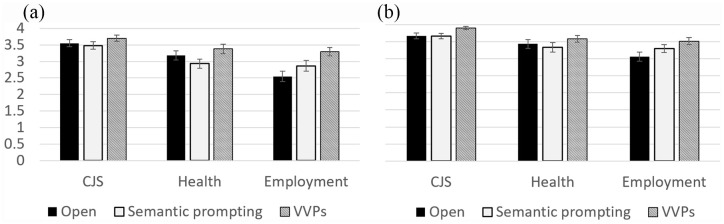
Mean specificity of responses by the (a) autistic group and (b) TD group, by support type and reporting context (error bars represent 95% confidence intervals).

There was also a Support × Context interaction, *F*(3.21, 179.70) = 4.80, *p* = .002, $\eta_{p}^{2}$ = .08. Within-subject contrasts indicated that, compared to open questions, semantic prompting resulted in decreased specificity for the health context, but increased specificity for the employment context (*p* = .008, $\eta_{p}^{2}$ = .12). Moreover, compared to open questions, V-VP improved specificity for the employment context to a greater extent than the CJS, *p* = .003, $\eta_{p}^{2}$ = .14, and health contexts, *p* = .002, $\eta_{p}^{2}$ = .16. There were no Group × Support, *F*(2, 112) = 0.68, *p* = .505, $\eta_{p}^{2}$ = .01; Group × Context, *F*(2, 112) = 1.96, *p* = .145, $\eta_{p}^{2}$ = .03; Group × Prep, *F*(1, 56) = 1.90, *p* = .174, $\eta_{p}^{2}$ = .03; or Group × Support × Context interactions, *F*(4, 224) = 0.72, *p* = .582, $\eta_{p}^{2}$ = .01. Therefore, autistic adults’ responses were less specific overall, but questioning support improved performance for both the autistic and TD groups (Figure 1).

### Relevant and irrelevant episodic and semantic information

The proportion of episodic relevant, episodic irrelevant, semantic relevant and semantic irrelevant details were calculated as a function of each participant’s total recalled details.

#### Proportion of relevant episodic detail

There were no main effects of Group, *F*(1, 56) = 2.69, *p* = .107, $\eta_{p}^{2}$ = .46, or Prep, *F*(1, 56) = 0.01, *p* = .926, $\eta_{p}^{2}$ < .001. There was a main effect of Support, *F*(2, 112) = 10.59, *p* < .001, $\eta_{p}^{2}$ = .16, whereby responses comprised a higher proportion of relevant episodic information with V-VP support (*M* = 0.76, *SD* = 0.14), compared to open questions, *M* = 0.71, *SD* = 0.15; *p* = .001, *d* = 0.34, and semantic prompting (*M* = 0.70, *SD* = 0.16; *p* < .001, *d* = 0.40), with no difference between open questions and semantic prompting, *p* = .660, *d* = 0.06. There was also a main effect of Context, *F*(2, 112) = 52.62, *p* < .001, $\eta_{p}^{2}$ = .48, whereby the CJS context yielded a higher proportion of relevant episodic details (*M* = 0.80, *SD* = 0.11) compared to the health (*M* = 0.71, *SD* = 0.17, *p* < .001, *d* = 0.63) and employment contexts (*M* = 0.64, *SD* = 0.17; *p*s < .001, *d* = 1.12). Finally, there was a Support × Context interaction, *F*(4, 224) = 6.86, *p* < .001, $\eta_{p}^{2}$ = .11. For the employment context, participants particularly struggled to produce relevant episodic details with open questions and benefitted from semantic prompting, whereas answers to the CJS and health contexts did not benefit from semantic prompting, *F*(1, 56) = 5.22, *p* = .026, $\eta_{p}^{2}$ = .09. Moreover, V-VP (compared to open questions) improved episodic relevance for the employment context to a greater extent than the CJS context, *p* < .001, $\eta_{p}^{2}$ = .28, and the health context, *p* < .001, $\eta_{p}^{2}$ = .20. No other interactions were significant (all *F*s < 2.02, *p*s > .092 and $\eta_{p}^{2}s$ < .04). Thus, in contrast to the findings regarding specificity, autistic and TD adults produced similar proportions of relevant episodic detail, but in line with the effects on specificity, questioning support improved performance for both groups.

#### Proportion of relevant semantic detail

**Figure 2.**
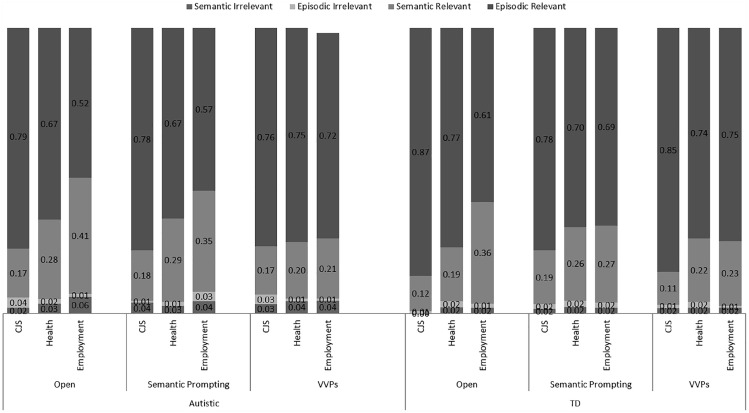
Proportion of relevant and irrelevant episodic and semantic details by support and context in the autistic and TD groups.

There was no main effect of Group, *F*(1, 56) = 1.46, *p* = .232, $\eta_{p}^{2}$ = .02, or Prep, *F*(1, 56) = 0.59, *p* = .447, $\eta_{p}^{2}$ = .01. There was a main effect of Support, *F*(2, 112) = 14.70, *p* < .001, $\eta_{p}^{2}$ = .21, with the proportion of relevant semantic information recalled declining with V-VP support (*M* = 0.19, *SD* = 0.10) compared to open questions (*M* = 0.25, *SD* = 0.12; *p* < .001, *d* = 0.54) and semantic prompting (*M* = 0.26, *SD* = 0.14; *p* < .001, *d* = 0.58), with no difference between open questions and semantic prompting (*p* = .874, *d* = 0.08). There was also a main effect of Context, *F*(2, 112) = 56.66, *p* < .001, $\eta_{p}^{2}$ = .50. Responses in the employment context comprised the highest proportion of relevant semantic detail (*M* = 0.30, *SD* = 0.14), compared to the health (*M* = 0.24, *SD* = 0.14; *p* < .001, *d* = 0.43) and the CJS contexts (*M* = 0.16, *SD* = 0.09; *p* < .001, *d* = 1.19). Finally, there was a Support × Context interaction, *F*(3.05, 170.58) = 6.66, *p* < .001, $\eta_{p}^{2}$ = .11. For the employment context, semantic prompting reduced the proportion of relevant semantic information reported, whereas semantic prompting increased the proportion of relevant semantic detail for the health and CJS contexts, *p* = .017, $\eta_{p}^{2}$ = .10. No other interactions were significant (*F*s < 2.15, *p*s > .091, $\eta_{p}^{2}s$ < .04; see Figure 2).^4^

#### Proportion of episodic irrelevant detail

There were no main effects of Support, *F*(2, 112) = 0.32, *p* = .730, $\eta_{p}^{2}$ = .01, Context, *F*(2, 112) = 0.31, *p* = .736, $\eta_{p}^{2}$ = .01, or Prep, *F*(1, 56) = 0.73, *p* = .398, $\eta_{p}^{2}$ = .01, and no interactions (*F*s < 2.86, *p*s > .062, $\eta_{p}^{2}s$ < .05).

#### Proportion of semantic irrelevant detail

Autistic participants reported more irrelevant semantic information overall (*M* = 0.04, *SD* = 0.05) compared to TD participants (*M* = 0.02, *SD* = 0.03), *F*(1, 56) = 4.07, *p* = .048, $\eta_{p}^{2}$ = .07. There were no main effects of Support, *F*(1.69, 94.70) = 0.57, *p* = .570, $\eta_{p}^{2}$ = .01, Context *F*(1.80, 100.68) = 3.03, *p* = .052, $\eta_{p}^{2}$ = .05, or Prep *F*(1, 56) = 0.82, *p* = .368, $\eta_{p}^{2}$ = .01, and no interactions (*F*s < 1.92, *p*s > .123, $\eta_{p}^{2}s$ < .03).

### Qualitative analysis of participant feedback

Participants’ responses to questions about their experience of the interview were analysed using content analysis (Mayring, 2015), coding responses within main themes and subthemes. The first author independently developed the codes and coded all data. A second rater then coded the data. The first author and second rater met to discuss discrepancies in codes and decide on final codes before the first author applied the final coding template to the entire dataset (see Supplementary Materials H).

#### Perceptions of question support

All interviewees provided feedback with regard to their preferred question type, things they found easy/difficult and why (see Table 3 for themes). Some participants explicitly stated that they found the open questions more difficult, due to a lack of guidance and difficulty gauging the appropriate level of detail. 13 participants commented on the semantic prompting being easy, but six found it challenging. Overall, both groups indicated a preference for V-VP prompting (30 interviewees), indicating that V-VPs enabled them to check the ‘completeness’ of their recall, acted as a general memory aid and visual cue, and improved the relevance and detail of their responses. A minority of participants indicated difficulties with V-VP (e.g. feeling that they were required to use every prompt and not knowing how, confusing the order of the elements, or forgetting to use the visual cue).

**Table 3.** Themes from participants’ responses about their perceptions of the questioning support.

Themes	ASD	TD	Example quotes
Open questions			I couldn’t organise my thoughts properly (Autistic participant) . . . I thought that’s a bit of an open question and that’s something I do struggle with (Autistic participant)
Difficult			
Difficult (general)	4	1	
Lack of guidance	1	2	
Unsure of level of detail	0	2	
Preferred/easiest			
Preferred/easiest (general)	4	5	
Semantic prompting			
Difficult			. . . always find it like, awkward, like, ’cause it’s not a conversation. So it’s just a bit strange and robotic . . . They just ask you a question, you answer and then they ask you a question and you answer. (Autistic participant) . . . was a bit . . . easier because that’s . . . sort of set me up to remember um how I feel about certain things. (Autistic participant)
Difficult (general)	2	4	
Preferred/easiest			
Preferred/easiest (general)	6	7	
V-VPs			
Difficult			I guess remembering to use this, I did forget that a couple of times. (Autistic participant) . . . that’s easier in some respects but then it puts pressure on you’re trying to think of something to fit that box. It’s like that you feel like you’ve got to complete something, finish it. It’s like there is something missing if you haven’t got it all there. So although it’s good to have the visual it can . . . put pressure on as well. (Autistic participant) . . . it’s just here on a plate for you, ’cause it kind of has everything that you need to talk about and everything you need to know about what you need to include in your answer, so I think that helped me quite a bit. (Autistic participant) I think I preferred using the prompt cards it gave me sort of the . . . way to sort of space out my sentences. (Autistic participant) It was easier having that, having the visuals and having something there . . . That helped me focus. (Autistic participant)
Difficult (general)	2	3	
Difficulties in addressing each point	4	2	
Difficulties with the order of prompts	1	1	
Feeling pressure to fulfil all aspects	2	1	
Preferred/easiest			
Preferred/easiest	14	16	
Organisation			
Help with structure	1	7	
Completeness	2	2	
Memory aid			
Visual cue	4	6	
Helps recall	3	4	
Prompted relevance	2	2	
Prompted detail	2	4	

ASD: autism spectrum disorder; TD: typically developing; V-VP: visual–verbal prompting.

#### Perceptions of the preparation condition

As seen in Table 4, although one autistic participant indicated that they would have preferred not to have received preparation (‘I’d rather go in cold’), most participants indicated its value in feeling prepared for the interview.

**Table 4.** Themes from participants’ responses about their perceptions of the preparation condition.

Themes	ASD	TD	Example quotes
Prep useful			
Prep useful (general comments)	3	8	It was fine because I could then think about it when I was at home, which I find things easier at home. (Autistic participant) . . . (making notes) certainly helped me keep to topic a little bit. (Autistic participant) . . . notes were useful . . . so I could focus on one specific thing ’cause sometimes my brain can go through like 50 different thoughts at the same time. So it helped me like focus on that one thing. (Autistic participant) . . . it takes me a while to . . . search through my memories and to find a specific um thing, but once I know er about it, I can quickly think back to that and to, um to remember it um, so without the preparation questions I would’ve had to, think for a long time before I remembered each individual event. (Autistic participant)
Would have been difficult without prep	3	5	
Making notes helpful	9	12	
Reduced anxiety	1	0	
Memory aid			
General memory prompt benefits	2	4	
Had examples ready	4	2	
Would need more thinking time without preparation	6	6	
Avoided over-preparing	1	1	
Effects on support			
Support did not differ/conflicted	2	1	
Changed recall (in semantic prompting condition)	0	1	
Changed recall	1	1	
Prep not useful			
Making notes unhelpful	1	0	
Prep unhelpful	1	0	

ASD: autism spectrum disorder; TD: typically developing.

## Discussion

The current study tested the efficacy of two novel methods of questioning support (semantic prompting and V-VP) in improving the specificity and relevance of ABM recall by autistic and TD participants in CJS, health and employment contexts, compared to standard open questioning. Consistent with predictions, responses from autistic participants were less specific overall than TD participants. Nevertheless, V-VP support improved specificity and increased the proportion of relevant episodic information reported by both groups. In contrast to predictions, autistic participants’ responses did not contain a lower proportion of relevant episodic (or semantic) detail compared to TD participants. They did, however, comprise more semantic irrelevant detail, thus partially supporting our prediction regarding relevance. No significant quantitative effects of preparation were found.

That autistic participants’ responses were of lower specificity than TD participants provides further evidence for the over-generality of ABMs in autistic adults (e.g. Adler et al., 2010; Chaput et al., 2013; Crane et al., 2009, 2012; Crane & Goddard, 2008; Tanweer et al., 2010). However, there were no significant differences between groups in terms of the proportion of episodic (relevant or irrelevant) details reported. The present study included very detailed instructions (including a printout) even for the open questioning condition, which may have been sufficiently supportive to elicit comparable levels of episodically relevant detail from both groups (see also Losh & Capps, 2003; Losh & Gordon, 2014). Although autistic participants recalled more irrelevant semantic details than TD participants, this was a relatively small effect, with overall analyses indicating similar effects of support and context for both groups.

Compared to open questions and semantic prompting, more detailed and explicit questioning using V-VPs resulted in an overall improvement in specificity (as well as episodic relevance) for both autistic and TD groups, supporting the utility of the task support hypothesis (Bowler et al., 1997, 2004) in more applied settings, in line with previous findings within the CJS context (e.g. Almeida et al., 2019; Maras et al., 2013; Mattison et al., 2018; McCrory et al., 2007). The explicit V-VP prompts may reduce demands on relational retrieval processes (known to be a source of difficulty for autistic people; see Gaigg & Bowler, 2018), which would typically aid the reconstruction of the event’s narrative with relations between specific details (who did what, to whom, where, when, etc.). V-VPs may also reduce implicit task demands, alleviating the need to infer what and how much to recall, in contrast to open questions (see Kenworthy et al., 2008; Müller et al., 2008; White et al., 2009).

Our findings highlight the importance of considering context. While semantic prompting did not improve specificity or episodic relevance overall across contexts, it was effective for the employment questions (albeit not to the same extent as V-VP), supporting previous findings by Robinson et al. (2017). Open questions may be particularly problematic in eliciting specific responses in an employment context. Semantic prompting may therefore be an effective method to support recall in contexts requiring the interviewee to relay personal characteristics and specific examples evidencing these (e.g. employment and promotion interviews). Although previous studies have found that autistic adults may not use the self to regulate ABM recall *spontaneously* (i.e. they do not appear to have a tendency to do so; Crane et al., 2009, 2010), our findings regarding the utility of semantic prompting for employment-related questions indicate that autistic people can use the self-memory system for episodic recall when they are explicitly instructed to do so. For the health context, however, semantic prompting decreased specificity.

Context-specific support effects are perhaps to be expected. When answering questions in an employment interview, we are usually thinking about ourselves (e.g. our personality and attributes) which may facilitate access to relevant specific memories (e.g. examples of acting upon these values). The autobiographical self-memory system implicates current goals of the working self in determining which events are remembered and ultimately accessible for recall (Conway & Pleydell-Pearce, 2000). Due to the nature of the CJS questions, semantic prompts were limited to personal preferences (e.g. ‘do you enjoy going to the supermarket?’), which may not be as effective in accessing semantic ABM compared to personal characteristics, which may be more easily linked to goals of the working self (Conway & Pleydell-Pearce, 2000). Furthermore, although semantic prompts for the health context also utilised personal attributes (e.g. ‘are you clumsy?’), these contexts may lend themselves more naturally to specific events (e.g. falling over as a discrete event). According to Conway and Pleydell-Pearce (2000), emotional cues are generally the least effective in prompting autobiographical recall, and people retrieve more memories associated with mild positive affect compared to intense positive or to negative emotions. In this study, to prompt memories related to mental health, the semantic prompts could be categorised as mild negative emotional cues (e.g. ‘are you a worrier?’). Since people tend to inhibit the recall (and, crucially, the re-experiencing) of negative emotions, especially when these are incongruous to the perceived self (e.g. perceived negative connotations of being a worrier), such prompts may limit the autonoetic awareness required to recall detailed episodic memories (Conway et al., 1997; Tulving, 1985; Wheeler et al., 1997). Indeed, participants noted that memories related to emotions were often difficult to recall (a feedback point endorsed more often by autistic than TD participants).

Qualitative analysis of participant feedback provides further evidence that the open questions were the most difficult, with a clear preference for V-VPs, and mixed responses regarding semantic prompting. Participants indicated the usefulness of V-VPs in providing a general aid for memory and a useful visual cue, as well as in specifying the amount and relevance of detail required. A minority of participants in both groups, however, commented that V-VP questions could be difficult due to needing to remember to refer to them, a desire to fulfil all criteria and a feeling of not being able to do so effectively in some cases (i.e. depending on the content of the question). This emphasises the importance of tailoring support to the context (e.g. in order to be effective in a CJS context, V-VPs would need to focus on aspects including who did what, to whom, where and when).

The absence of quantitative effects of preparation may be due to the already very detailed interview instructions, whereby the type of detail to include was clearly specified (with a comprehensive example), and participants being prompted when their answers were not clearly relaying a specific event (which, although analysed separately, may nonetheless have induced an order effect). Nonetheless, participants generally reported that preparation was helpful (e.g. in reducing thinking time). Preparation may be a particularly valuable tool for reducing anxiety in police and employment interviews, and in healthcare consultations.

Limitations of the current study are acknowledged. Clearly, ideal answers to interview questions in different contexts vary; whereas questioning in CJS and health contexts often focuses on specific events, the interviewee should be ‘selling themselves’ in an employment interview (and focusing on one specific instance may not always be an effective strategy). As the current study focused on investigating effective methods to support recall, it was not possible to capture all differences between applied contexts within a single design; however, this is an important area for future research. Relatedly, our findings from the frequency survey conducted to inform the ABM interview questions merit further investigation. TD participants reported engaging in CJS-context (e.g. going to the supermarket, cinema) and employment-context (e.g. working in a team, being organised) activities more frequently than the autistic group, whereas the autistic group reported a higher frequency of health-context experiences than the TD group. The effect of these disparities in experience on recall should be investigated in future. Finally, although the groups in the current study were matched on age and IQ, it was not possible to match the groups on sex. Future research should aim to match the groups on sex, as some sex differences are found in ABM (Grysman & Hudson, 2013; Herlitz & Rehnman, 2008; Schulkind et al., 2012), although the findings regarding sex differences for relevance and specificity are mixed (Baron & Bluck, 2009; Bluck et al., 2005; Wang, 2004).

In conclusion, the current findings demonstrate how flexibly employing different methods of questioning support may be valuable in supporting recall by autistic and TD people in different contexts. V-VP may be universally useful in minimising task ambiguity and freeing up cognitive resources to elicit an appropriate strategy for memory searching, with potential added value in using semantic prompting in employment and related contexts. Further, V-VP may be particularly useful in police interviews and is somewhat analogous to the five-part statement structure used by police in obtaining written statements (i.e. introduction, people, places, ‘what happened’ and descriptions, for example, people/property).

## Supplemental Material

AdaptingInterviews_SupplementaryMaterials2 – Supplemental material for Interviewing autistic adults: Adaptations to support recall in police, employment, and healthcare interviews Click here for additional data file. Supplemental material, AdaptingInterviews_SupplementaryMaterials2 for Interviewing autistic adults: Adaptations to support recall in police, employment, and healthcare interviews by Jade Eloise Norris, Laura Crane and Katie Maras in Autism

## Notes

1. The responses for the autistic and typically developing (TD) groups were summed as frequencies for each context (criminal justice system (CJS), health, employment), with group (autistic vs TD) as a between-subjects factor. A repeated-measures analysis of variance (ANOVA) indicated no main effect of Group, *F*(1, 119) = 0.01, *p* = .956, $\eta_{p}^{2}$ < .01. There was a main effect of Context, *F*(2, 238) = 53.03, *p* < .001, $\eta_{p}^{2}$ = .31, with a higher reported frequency of activities in the employment (*M* = 9.02, *SD* = 2.05) than the health context (*M* = 7.20, *SD* = 1.87; *p* < .001, *d* = 0.93), for health compared to CJS activities (*M* = 6.87, *SD* = 1.28; *p* = .007, *d* = 0.21) and for employment compared to CJS activities (*p* < .001, *d* = 1.26). There was a Group × Context interaction, *F*(2, 238) = 8.93, *p* < .001, $\eta_{p}^{2}$ = .07. Within-subject contrasts indicate that the TD participants reported engaging in CJS-related activities (going to the supermarket, etc.) more frequently than the autistic group, whereas the autistic group reported engaging in more health-related behaviours/activities (feeling worried, falling over, etc.) than the TD group, *p* = .001, $\eta_{p}^{2}$ = .09. Moreover, the TD group reported engaging in employment-related activities (working in a team, etc.) more frequently than the autistic group, *p* < .001, $\eta_{p}^{2}$ = .12.

2. A univariate ANOVA, 2 (Group: autistic vs TD) × 2 (Prep: prep vs no prep), was conducted on the mean duration of the interviews. There were no significant main effects nor interactions (*p*s > .352).

3. A mixed factorial ANOVA, 2 (Group: autistic vs TD) × 2 (Prep: prep vs no prep) × 3 (Support: open vs semantic prompting vs visual–verbal prompting (V-VP)) × 3 (Context: CJS vs health vs employment), with Support and Context as within-subject factors, was conducted on the total number of prompts required when participants were not relaying a specific instance. The number of prompts given declined with Support, *F*(2, 112) = 12.67, *p* < .001, $\eta_{p}^{2}$ = .19: open questions resulted in the highest number of prompts (*M* = 0.17, *SD* = 0.15), followed by semantic prompting (*M* = 0.11, *SD* = 0.14), with the lowest number of prompts needed for V-VP (*M* = 0.05, *SD* = 0.10; *p*s < .05). A main effect of context, *F*(1.54, 86.40) = 36.03, *p* < .001, $\eta_{p}^{2}$ = .39, indicated that the number of prompts required was higher for the employment context (*M* = 0.22, *SD* = 0.20) compared to the health context (*M* = 0.09, *SD* = 0.12; *p* < .001, *d* = 0.79) and to the CJS context (*M* = 0.02, *SD* = 0.06; *p* < .001, *d* = 1.35). Finally, a Support × Context interaction, *F*(2.92, 163.25) = 9.75, *p* < .001, $\eta_{p}^{2}$ = .15, indicated that, whereas semantic prompting decreased the need for prompting for the employment context compared to open questioning, prompts increased for the health context with semantic prompting compared to open questioning (*p* < .001, $\eta_{p}^{2}$ = .21). Furthermore, while V-VP support reduced the number of prompts required for the health context compared to semantic prompting, the number of prompts remained the same for the CJS questions with semantic prompting and V-VP (i.e. lower than all other contexts across all levels of support, *p* = .004, $\eta_{p}^{2}$ = .14). There was no main effect of Group (*p* = .433, $\eta_{p}^{2}$ = .01) or Prep (*p* = 1.00, $\eta_{p}^{2}$ = .00).

4. There were no effects or interactions when the proportion of *overall relevant* and *overall irrelevant* information (episodic and semantic) was combined (*p*s > .091).
