# Supplementary material for: Interviewing autistic adults: Adaptations to support recall in police, employment, and healthcare interviews
Source: Autism. 2020 Mar 23;24(6):1506–20. doi: 10.1177/1362361320909174 (PMC7376628; doi:10.1177/1362361320909174)
Supplement: AdaptingInterviews_SupplementaryMaterials2 – Supplemental material for Interviewing autistic adults: Adaptations to support recall in police, employment, and healthcare interviews [file AdaptingInterviews_SupplementaryMaterials2.pdf]

Supplementary materials A: Question topics within each Support x Context condition

	Open	Semantic	V-VP
**CJS (functional activities)**	Train (bus)	Supermarket	Bank (Post office)
**CJS (leisure activities)**	Cinema (theatre)	Party	Out for meal
**Health (Physical)**	Vomited	Fallen over/ bashed into something	Accidentally cut yourself
**Health (Mental)**	Angry	Worried	Sad
**Employment interview (Social)**	Worked as a team	Disagreement	Use technology
**Employment interview (Non-social)**	Difficult decision	Organising	Deadline

Supplementary materials B: General instructions for the ABM interview

Hi, thanks again for taking part in this study. There will be three blocks of questions about memory, and we can have a short break in between the blocks if we need to. Try to answer as best you can, but there aren't any right or wrong answers. As the interviewer, I'm only allowed to say certain things in response to your answers.

So, I'll be asking you to remember and tell me about some things that you have personally experienced. When I ask you the questions, I'd like you to tell me a specific memory that you have – a memory of a particular event lasting no longer than a day – from more than a week ago (so don't recall something that happened within the last 7 days) but it can be any memory from before this.

We're interested in your ability to recall in detail a specific instance each time, so try to give details of an event, or things that happened at only one time, at a particular place and within the same, single day. You should try to include the 'who, what, where and when' of the memory, and try to recall a different instance/event for each question.

Here's an example of a specific memory for going swimming: "I remember it was during summer but it was a really cold day. My brother insisted we try out the new outdoor pool at the leisure centre. I remember I had my new red swimming trunks on. There was hardly anyone at the pool, and I remember jumping in and the cold of the water taking my breath away! I got out as quickly as I could and my brother called me wimp".

Finally, take your time to think about the memory before giving your answer whenever you need to. These instructions are here for you if you need them.

Supplementary materials C: Participant printed notes provided at interview

- Tell me a *specific* memory
    - from *more* than a week ago
    - try to give details of an event, or things that happened within one day

- There aren't any right or wrong answers

- Take your time to think about the memory before answering

Supplementary materials D: Final Questions (at the end of the ABM interview)

1. Was there anything you found particularly difficult?
2. Anything you found easy?
3. Which block of questions did you prefer/find easier to answer?
4. Is there anything that we didn't do that would have made it easier for you to answer the questions?
5. Any other thoughts?

IF in prep condition =

1. Did you find the preparation information useful?
2. (did you make and bring notes?/ I see you've brought some notes) did you find this helpful?

Supplementary materials E: specificity coding (as in Piolino, Desgranges, Benali, & Eustache, 2002)

**Score and criteria**	**Example response**
4 = Specific event (isolated, situated in time and space) with rich detail in terms of actions, thoughts, perceptions, images, etc.	*"I went to the bank two weeks ago, on a Thursday. It was sunny and a nice lady with long hair greeted me at the counter. I asked her if I could discuss a mortgage application, so she showed me upstairs to a little private booth. I found it odd that they had glass windows all the way around, and all of the booths had the same three pictures on the wall…"*
3 = Specific event (isolated, situated in time and space) with few details	*"I went to the bank two weeks ago, on Thursday. I didn't have to wait long so it was all over pretty quickly."*
2 = Generic event (repeated or prolonged over time, situated in time and space)	*"I go to the bank every Thursday to pay in cash for work. Last time was just the same as ever."*
1 = Vague event (repeated or prolonged over time, not situated in time and space),	"*Whenever I go it's always so busy. I avoid it at all costs."*
0 = Absence of memory	"*I don't know"* or "*I can't think of anything"* or general information about a theme, e.g., *"I never go, I hate banks"*

Supplementary materials F: Example response coded for relevance

In this example response: *"I walked into the bank. It was the bank on Broad Street. I usually avoid going to the bank. Like the other time when I went to a different branch and it was terrible. I also avoid supermarkets"*, "walked", "bank", and "broad street" would be coded as relevant episodic details, "I usually avoid going to the bank" would be coded as a relevant semantic detail, "Like the other time when I went to a different branch" and "it was terrible" would be coded as irrelevant episodic details, and "I also avoid supermarkets" would receive a semantic irrelevant code.

Supplementary materials G: Analyses including responses after the 24 hour prompt

Specificity of responses

A 2 (Group: autistic vs. TD) x 2 (Prep: prep vs. no prep) x 3 (Support: Open vs. Semantic Prompting vs. V-VP) x 3 (Context: CJS, health, employment) mixed factorial ANOVA was conducted for overall mean specificity as scored with data *including* the 24-hour prompt (support and context were within-subjects factors). There was a main effect of support, $F(2,112) = 14.37$, $p < .001$, $\eta p^2 = .20$. Pairwise comparisons indicated significantly higher specificity for responses to V-VP ($M = 3.63$, $SD = 0.40$) compared to open questions ($M = 3.40$, $SD = 0.53$) and to semantic prompting (M = 3.38, SD = 0.50),$ps < .001$, with no difference in specificity between open questions or semantic prompting ($p = .738$). There was also a main effect of context, $F(2,112) = 24.27$, $p < .001$, $\eta p^2 = .30$. Pairwise comparisons indicated that specificity was higher for responses to questions in the CJS context ($M = 3.66$, $SD = 0.40$) compared to health ($M = 3.41$, $SD = 0.50$) and employment ($M = 3.34$, $SD = 0.53$), $ps < .001$, with no difference in specificity between the health and employment contexts ($p = .195$). There was also a main effect of group, $F(1,56) = 6.39$, $p = .014$, $\eta p^2 = .10$. Autistic participants produced memories with overall lower specificity ($M = 3.34$, $SD = 0.45$) compared to the TD group ($M = 3.60$, $SD = 0.35$). There was no main effect of Prep, $F(1, 56) = .428$, $p = .516$, $\eta p^2 = .01$. There was a Group x Context interaction, $F(1, 112) = 3.38$, $p = .037$, $\eta p^2 = .06$. Within-subjects contrasts indicate that whereby the autistic group's specificity declined for the employment context compared to the health context, the TD group had similar responses for both of these contexts ($F(1,56) = 6.51$, $p = .013$, $\eta p^2 = .10$). There was also a three-way interaction between Support, Context, and Group, $F(4, 224) = 3.38$, $p = .010$, $\eta p^2 = .06$.

Relevant and irrelevant episodic and semantic information

A further four 2 (Group: autistic vs. TD) x 2 (Prep: prep vs. no prep) x 3 (Support: Open vs. Semantic Prompting vs. V-VP) x 3 (Context: CJS, health, employment) mixed factorial ANOVAs were conducted for the data including the 24-hour prompt for the proportion of: episodic relevant, episodic irrelevant, semantic relevant, and semantic irrelevant information produced, respectively (support and context were within-subjects factors).

Proportion relevant episodic detail. There was a main effect of Support, $F(2,112) = 8.55$, $p < .001$, $\eta p^2 = .13$. Pairwise comparisons indicated a significantly higher proportion of episodic relevant detail for responses to V-VP ($M = .766$, $SD = 0.14$) compared to open questions ($M = .727$, $SD = 0.15$), $p = .006$ and compared to semantic prompting (M = .708, SD = 0.15), $p < .001$, with no difference in the proportion of episodic relevant information between open questions and semantic prompting ($p = .254$). There was also a main effect of Context, $F(2,112) = 39.40$, $p < .001$, $\eta p^2 = .41$. Pairwise

comparisons showed that the proportion of episodic relevant details was higher for the CJS context (M = .803, SD = 0.11) compared to health (M = .725, SD = 0.16) and compared to employment (M = .673, SD = 0.17; ps > .001), and for the health context compared to employment (p = .002). There was no main effect of Group, $F(1,56) = 3.20$, $p = .079$, $\eta p^2 = .05$. Finally, there was a Support x Context interaction, $F(3.50, 195.83) = 5.21$, $p = .001$, $\eta p^2 = .09$. Within-subjects contrasts indicated an increased proportion of episodic relevant details for the employment context with semantic prompting, compared to a decrease in the proportion of episodic relevant details for the CJS and health contexts with semantic prompting, $F(1,56) = 4.39$, $p = .041$, $\eta p^2 = .07$.

Proportion relevant semantic detail. There was a main effect of Support, $F(2,112) = 10.77$, $p < .001$, $\eta p^2 = .16$, with a significantly higher proportion of semantic relevant detail for open questions (M = .23, SD = 0.12) compared to V-VP (M = .186, SD = 0.10; $p < .001$), and for semantic prompting (M = .24, SD = 0.13) compared to V-VP ($p < .001$) with no difference between open questions and semantic prompting ($p = .451$). There was also a main effect of Context, $F(1.80, 100.79) = 43.52$, $p < .001$, $\eta p^2 = .44$. Pairwise comparisons indicated that the proportion of semantic relevant details was highest for the employment context (M = .28, SD = 0.13) compared to the health (M = .23, SD = 0.12; $p = .002$) and CJS contexts (M = .16, SD = 0.09; $p > .001$), and for the CJS context compared to health ($p < .001$). There was no main effect of Group, $F(1,56) = 2.15$, $p = .148$, $\eta p^2 = .04$. Finally, there was a Support x Context interaction, $F(3.06, 171.28) = 5.06$, $p = .002$, $\eta p^2 = .08$. Within-subjects contrasts indicated a decreased proportion of semantic relevant details for the employment context with semantic prompting compared to open questions, whereas the proportion of semantic relevant details increased with semantic prompting for the CJS and health contexts, $F(1,56) = 4.60$, $p = .036$, $\eta p^2 = .08$. No other main effects or interactions were significant (all ps > .069).

Proportion episodic irrelevant detail. There was a Group x Context interaction, $F(2, 112) = 3.37$, $p = .038$, $\eta p^2 = .06$. Within-subjects contrasts indicated that this was due to the TD group producing an increased proportion of episodic irrelevant details for the health context compared to CJS, whereas the autistic group produced a lower proportion of episodic irrelevant details for the health context compared to CJS, $F(1,56) = 5.82$, $p = .019$, $\eta p^2 = .09$. No other main effects or interactions were significant (all ps > .111).

Proportion semantic irrelevant detail. There was a main effect of Context, $F(2, 112) = 4.18$, $p = .018$, $\eta p^2 = .07$. Pairwise comparisons indicated that this was due to a higher proportion of semantic irrelevant details for the employment context (M = .04, SD = 0.05) compared to CJS (M = .02, SD = 0.03; $p = .010$), whereas the differences between CJS and health (M = .03, SD = 0.05; $p = .142$), and employment and health ($p = .130$) were non-significant. No other main effects or interactions were significant (all ps > .051).

Supplementary materials H: Additional content analysis tables

Themes from participants' responses about aspects of the interview they found difficult

**Themes**	**ASD**	**TD**	**Example Quotes**
General difficulties (e.g., bad memory in general)	13	9	…um, remembering some of the, uh instances were difficult… my memories tend to get a little mixed up if its, if it's a recurring setting or, um, or event, so it was hard to separate them unless I got something that, attaches them all together in a specific event… [Autistic participant]<br><br>… that's something I do struggle with, a lot, is open ended questions. I'm always asking people to be more not so open ended… [Autistic participant]<br><br>Just sort of trying to get things, erm, together, erm, in my mind… sort of trying… to remember it all and that sort of thing, and trying to get memories into coherent sense really. [Autistic participant]
Remembering dates	2	1	
Gauging detail level or relevance	3	6	
Being unprepared	0	2	
Feeling uncomfortable about question topic	1	2	
Many questions	1	0	
Used bad example	0	1	
Some questions more difficult than others			
Some questions more difficult than others (general)	4	7	
Mundane or day-to-day difficult	6	5	
Emotion more difficult to recall	6	3	
Infrequent activity	0	1	
Dislike interview environment	1	0	

Themes from participants' responses about aspects of the interviewee they found easy

Themes	ASD	TD	Example quotes
General ease (good memory, easy to talk about self, etc)	9	18	I generally find talking about myself easy… so err that was fine [Autistic participant]<br><br>… I must admit, I did find it, strangely easy recalling things like um, the supermarket and also the restaurant, in Pizza Hut… especially the restaurant one, even though it was awkward at the time I sort of look back and sort of it just brings a slight smile to my face… [Autistic participant]<br>I think… what comes to light is emotional memories. Because you know, that's what really, you remember. It's those emotional times that suddenly point you in another direction in life. [TD participant]<br><br>I tend to remember things which had more of an emotional impact I suppose. [Autistic participant]
Liking the interview environment	0	1	
Some questions easier than others			
Some questions easier than others (general)	2	2	
Emotions easier to recall	1	2	
More recent memories easier	2	1	
Some Qs more specific, easier	2	0	
